# Simultaneous sleep study and nasoendoscopic investigation in a patient with obstructive sleep apnoea syndrome refractory to continuous positive airway pressure: a case report

**DOI:** 10.1186/1752-1947-3-9315

**Published:** 2009-12-02

**Authors:** Claudia Chaves Loureiro, Marta Drummond, Adriana Magalhães, Elisabete SantaClara, Miguel Gonçalves, João Carlos Winck

**Affiliations:** 1 Department of Pulmonology, University Hospital of Coimbra, Coimbra, Portugal; 2 Department of Pulmonology, São João do Porto Hospital, Porto, Portugal

## Abstract

**Introduction:**

The standard treatment for obstructive sleep apnoea syndrome is nasal continuous positive airway pressure. In most cases the obstruction is located at the oropharyngeal level, and nasal continuous positive airway pressure is usually effective. In cases of non-response to nasal continuous positive airway pressure other treatments like mandibular advancement devices or upper airway surgery (especially bi-maxillary advancement) may also be considered.

**Case presentation:**

We report the case of a 38-year-old Caucasian man with severe obstructive sleep apnoea syndrome, initially refractory to nasal continuous positive airway pressure (and subsequently also to a mandibular advancement devices), in which the visualization of the upper airway with sleep endoscopy and the concomitant titration of positive pressure were useful in the investigation and resolution of sleep disordered breathing. In fact, there was a marked reduction in the size of his nasopharynx, and a paresis of his left aryepiglotic fold with hypertrophy of the right aryepiglotic fold. The application of bi-level positive airway pressure and an oral interface successfully managed his obstructive sleep apnoea.

**Conclusion:**

This is a rare case of obstructive sleep apnoea syndrome refractory to treatment with nocturnal ventilatory support. Visualization of the endoscopic changes, during sleep and under positive pressure, was of great value to understanding the mechanisms of refractoriness. It also oriented the therapeutic option. Refractoriness to obstructive sleep apnoea therapy with continuous positive airway pressure is rare, and each case should be approached individually.

## Introduction

Obstructive sleep apnoea syndrome (OSAS) is characterized by a recurrent collapse of all or some parts of the upper airway during sleep. Despite being sub-diagnosed, it affects 2% to 4% of the world's population [1] and has a higher prevalence in obese people [2]. This syndrome is associated with increased cardiovascular risk. It is also an independent risk factor for hypertension, myocardial infarction and stroke [3].

The method for its initial evaluation using a cardio-respiratory study is simple and easy to use on an outpatient basis.

Nasal continuous positive airway pressure (nCPAP) during sleep, which allows airway patency, is the current standard treatment [4]. It significantly improves patients' excessive daytime sleepiness, states of wakefulness, cognitive abilities [5], and quality of life [6]. This treatment also decreases cardiovascular risk, especially when it is used for more than 4 hours daily [7].

Alternative treatments include a mandibular advancement device (MAD) that increases the lumen of the airway by inducing jaw and tongue protrusion during sleep, improves the tone of the muscles of the airway, and reduces the passive compliance of the pharyngeal wall [8]. It is especially effective in non-obese patients with moderate OSAS.

Upper airway surgery, specifically bi-maxillary surgery, is also effective in severe cases of OSAS. It may be considered for patients who are unwilling to use, or are refractory to, nCPAP therapy and whose anatomical changes are prone to surgical resolution [9]. This approach must be made and addressed specifically.

## Case presentation

We report a 38-year-old Caucasian man who was referred to our department for suspected OSAS with complaints of severe snoring, respiratory pauses that were witnessed by his wife, morning headaches, and adynamia, but without acknowledgement of excessive daytime sleepiness.

He had a history of dyslipidemia treated with diet and statin, without the existence of other cardiovascular risk factors. He had low alcohol consumption (10 gr/day) and no history of smoking. A physical exam revealed macroglossia, a bulky soft palate and uvula. He was overweight with a body mass index (BMI) of 29.1 and had a cervical perimeter of 42 cm. As an initial diagnostic approach, a spirometry and chest X-ray were performed, which revealed no changes. A diagnostic cardiorespiratory study showed that in addition to extended periods of snoring, he also had severe OSAS with an apnoea and hypopnoea index (AHI) of 72.1/h, a desaturation index of 67.1/h, and a minimum O2 saturation of 69%.

With the diagnosis of severe OSAS, despite the lack of excessive daytime sleepiness, a trial of positive airway pressure (automatic mode) was proposed, with the minimal pressure of 4 cmH20 and maximum pressure of 15 cmH2O. General measures of sleep hygiene and weight reduction were also recommended. As an alternative, the use of MAD was considered, and the patient was referred to our hospital's orthodontics department.

The patient was evaluated after 3 months and there was no adherence to treatment, with only 3 minutes of use per night, with a total number of 6 days of use. The patient attributed this to his difficulty in adapting to the masks and to the pressure itself.

**Figure 1:**
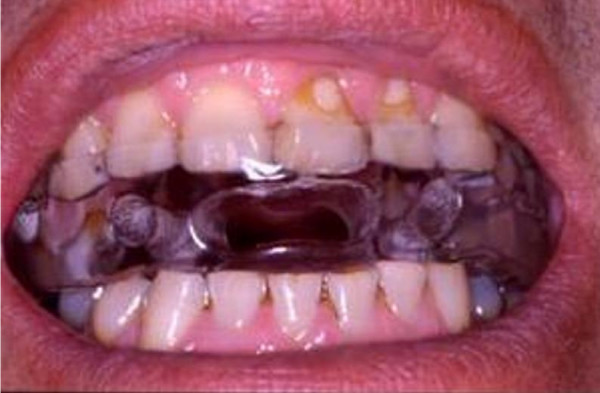
**An example of a mandibular advancement device**.

MAD (Figure 1) was applied over the next 3 months. During this period, our patient used the device daily for 3 to 4 hours per night, as limited by some salivation and gum pain. His clinical symptoms, however, did not improve.

**Figure 2:**
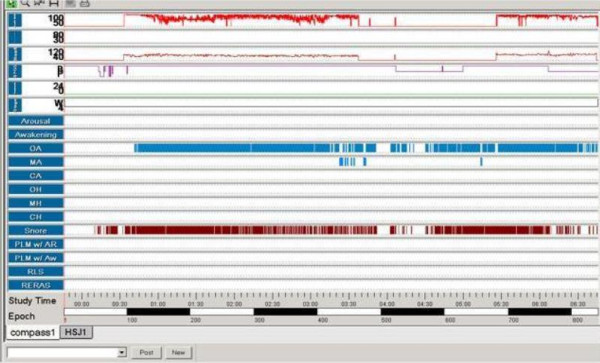
**A cardiorespiratory study in the use of a mandibular advancement device**. An evaluation at 4 months with a cardiorespiratory study in the use of a mandibular advancement device (first 3.5 h of study) showed no significant improvement in the patient's obstructive sleep apnoea syndrome (apnoea and hypopnoea index at 61.4/h and desaturation index of 42.1/h). A severe condition of obstructive sleep apnoea syndrome was observed with and without the use of the device.

For a better evaluation of our patient's clinical response, we did a home cardiorespiratory study using MAD (Figure 2), which showed no significant improvement in his OSAS. (He had an AHI of 61.4/h and desaturation index of 42.1/h with MAD during the first 3.5 hours of recording).

**Figure 3:**
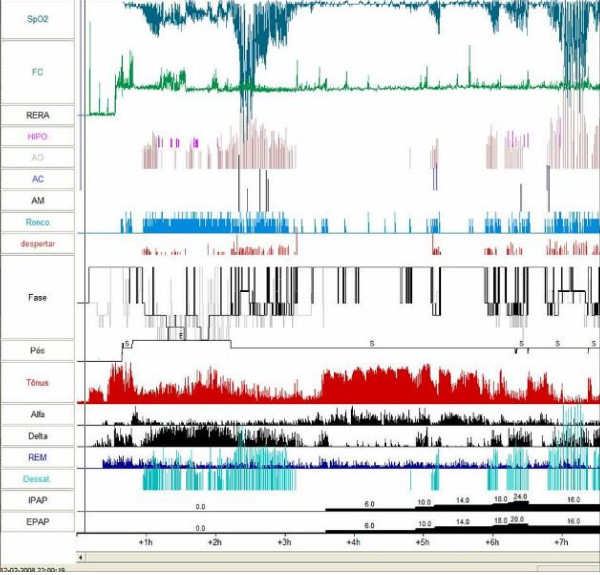
**Split-night polysomnography: Obstructive sleep apnoea syndrome refractoriness with continuous positive airway pressure and bilevel positive airway pressure, nasal mask**. (Evaluation at 4 months). The first part of the night confirmed the severity of obstructive sleep apnoea syndrome (apnoea and hypopnoea index of 64.9/h with minimum O2 saturation of 29%). The second part allowed a gradual increase in positive pressure, first in continuous positive airway pressure for up to 16 cmH2O, then in bilevel positive airway pressure with a maximum inspiratory pressure of 24 cmH2O and a maximum expiratory pressure of 20 cmH2O. There were persistent obstructive events with marked desaturation (minimum O2 saturation of 45% in continuous mode and of 82% in bilevel mode).

To titrate CPAP pressures and to better characterize our patient's sleep structure, we conducted a split-night polysomnography. The first part of the night confirmed the severity of our patient's OSA (AHI of 64.9/h with minimum O2 saturation of 29%). The second part allowed a gradual increase of positive pressure, first in continuous mode (CPAP) for up to 16 cmH2O, then in the bilevel mode (BiPAP) with a maximum inspiratory pressure (IPAP) of 24 cmH2O and a maximum expiratory pressure (EPAP) of 20 cmH2O. Persistent obstructive events with marked desaturation, with a minimum O2 saturation of 45% in CPAP mode and of 82% in BiPAP mode (Figure 3) were prevalent.

Since the nocturnal titration was ineffective, a retitration of pressures was conducted during the day to confirm this refractoriness and optimize the interface. At that moment the patient was prescribed bilevel-positive air pressure (VIVO 30, Breas) with 20 cmH2O of IPAP and 12 cmH2O of EPAP and a gel face mask (Mojo).

**Figure 4:**
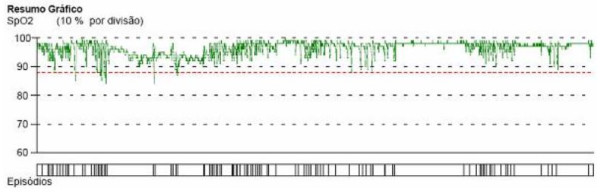
**A home nocturnal oximetry (in bilevel mode) with nasal mask**. (Evaluation at 5 months). A home nocturnal oximetry (in bilevel mode, inspiratory pressure of 20 cmH2O and expiratory pressure of 12 cmH2O, and a gel face mask (Mojo) shows episodes of desaturation suggesting a large number of residual apnoea and/or hypopnoea events.

After a period with these settings, the patient's symptoms remained, but he developed a newly diagnosed hypertension, which was treated with antihypertensive medication. Home nocturnal oximetry (in bilevel mode with those parameters) maintained episodes of desaturation, suggesting a large number of residual apnoea and/or hypopnoea events (Figure 4).

**Figure 5:**
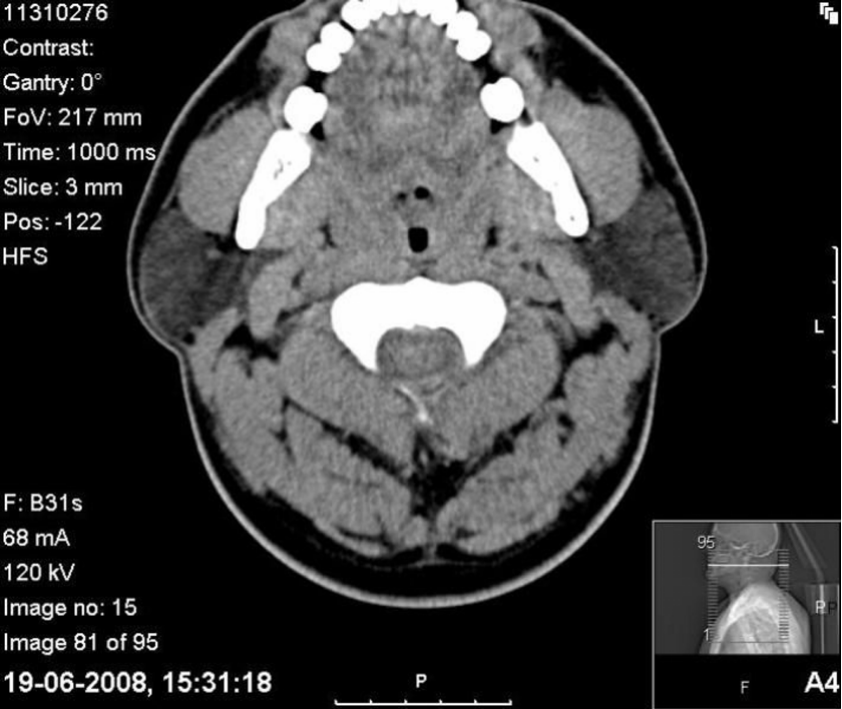
**A facial computed tomography at 5.5 months following the initial presentation shows a smaller upper airway**.

**Figure 6:**
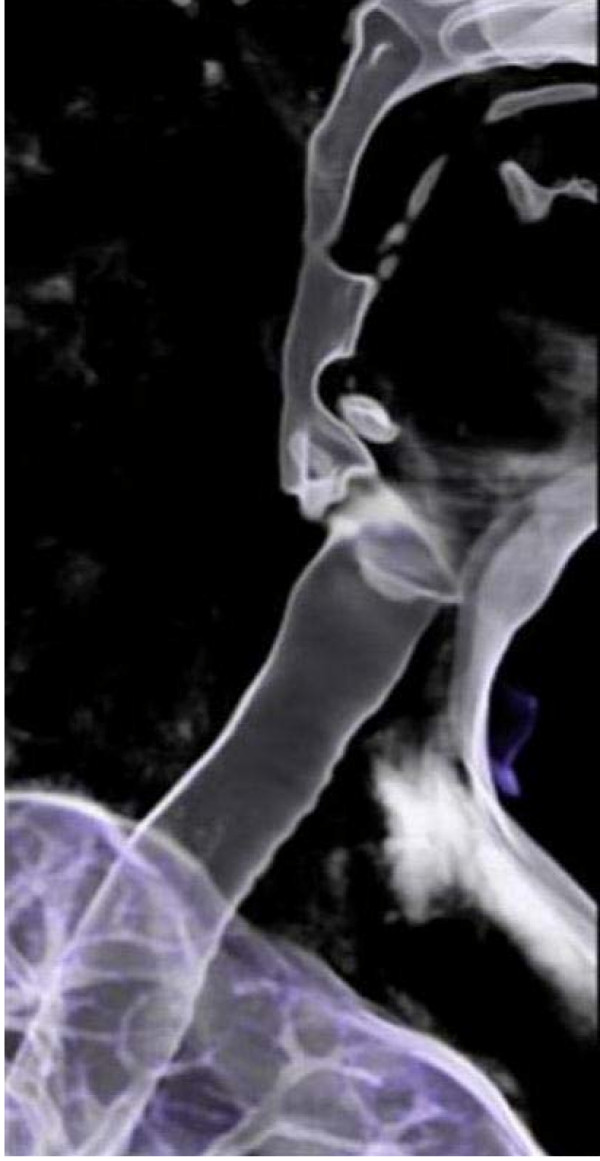
**A facial computed tomography reformation at 5.5 months after the initial presentation shows an angular dysmorphia at the hypopharynx level**.

His refractoriness led to further investigations which were done using two methods of evaluation. In the first one, a facial computed tomography (CT) revealed a smaller upper airway (Figure 5). Reformatting (Figure 6) showed an angular dysmorphia at the hypopharynx [10].

**Figure 7:**
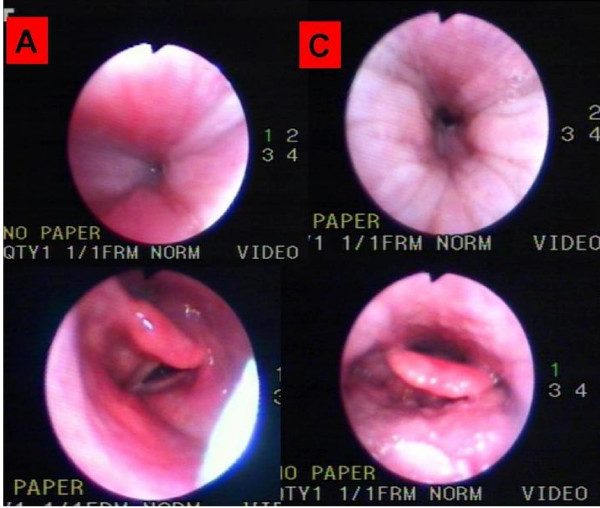
**(A) Sleep endoscopy before ventilation**. Extended periods of vibration of the walls of the oropharynx related to snores were observed. With the establishment of positive pressure ventilation, the nasopharynx subocclusion persisted up to 24 cmH2O inspiratory pressure and 16 cmH2O expiratory pressure. An unrolling of the epiglottis that collapsed the airway and provoked periods of O2 desaturation was also noted. **(C) **Sleep nasoendoscopy under continuous positive airway pressure with P > 16 cmH2O at 6 months after the initial presentation. In this evaluation, a marked reduction of the size of the nasopharynx, and a paresis of the left aryepiglotic fold with hypertrophy of the right one were noted.

**Figure 8:**
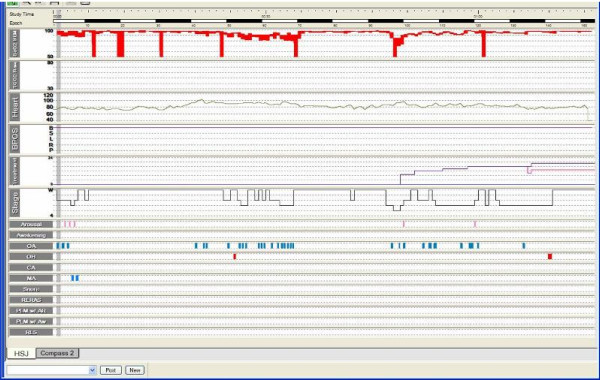
**A polysomnography study during sleep endoscopy at 6 months after the initial presentation**. A polysomnography and titration of ventilatory support pressures were also performed during sleep endoscopy.

**Figure 9:**
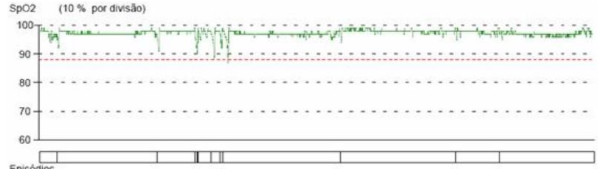
**A nocturnal oximetry under inspiratory pressure of 20 cmH20, expiratory pressure of 13 cmH20 with Oracle^® ^at 6.5 months after the initial presentation**. A home oximetry under bilevel mode, inspiratory pressure of 20 cmH20 and expiratory pressure of 13 cmH20 and Oracle^® ^mask revealed a significant improvement in nocturnal desaturation episodes.

The second method used sleep nasoendoscopy (Figure 7) with concomitant polysomnography and titration of ventilatory support pressures (Figure 8) [6]. In this evaluation, the patient was able to sleep effectively, and a marked reduction of the size of the nasopharynx and a paresis of the left aryepiglotic fold with hypertrophy of the right one (Figure 7A) were noted. Extended periods of vibration of the walls of the oropharynx related to snores were also observed. With the establishment of positive pressure ventilation, a subocclusion of the nasopharynx persisted (up to IPAP/EPAP levels of 24/16 cmH2O). An unrolling of the epiglottis that collapsed the airway and provoked periods of O2 desaturation (Figure 7C) was noted a few times. These episodes improved under 20 cmH20 IPAP and 13 cmH20 EPAP with an Oracle^® ^mask. A home oximetry under a bilevel mode with these pressures and interface (Figure 9) revealed a significant improvement in our patient's nocturnal desaturation episodes.

## Discussion

Refractoriness of OSAS therapy is rare and its approach should be targeted to specific individuals.

The conventional method for administering CPAP is using a nasal or oronasal interface [11] based on increasing intramural pressure above a critical point of oropharynx collapse (PCrit) [12]. Patients' compliance to treatment is somewhat constrained by the side effects associated with the use of these interfaces, such as nasal congestion, dryness of the oronasal mucosa, epistaxis, and claustrophobia. The oral route is an alternative that can be used in cases where the patient is intolerant to conventional approaches [13].

According to recent literature [14], the air acts as a resistor to the physiological nasal obstruction which produces collapsing forces that manifest at the most collapsible point, the pharynx. Positive pressure applied through the nose has to overcome the PCrit that results from the composition of pressure at the point of collapse of the airway and the surrounding soft tissue. Because the soft palate is complacent, the PCrit to be overcome is similar to the positive pressure that is applied through the mouth. The Oracle mask (Fisher and Paykel) has shown to be effective in the treatment of OSAS [13], as it applies a pressure-flow relationship to the oropharynx similar to that of the nasal way and imposes no obvious changes in the superior airway [15]. It also has the advantage of fewer side effects.

In this particular case, the visualization, during sleep and under positive pressure, of the endoscopic changes, was of great value to the understanding of the mechanisms of refractoriness.

The application of a positive pressure in an airway with anatomical changes (such as occurred in the case described) could perhaps have caused valve mechanisms that led to the unrolling of the epiglottis, with consequent obstruction to the passage of air. This phenomenon has become more evident with pressure levels greater than 16 cmH2O. At the same time, with lower pressures, the patency of the airway was not established.

Based on these findings, the clinical decision to administer bilevel positive pressure during sleep through an oral mask, which is not usually used in patients with OSAS, overcame the major collapse of our patient's nasopharynx.

## Conclusion

We describe a rare case of OSAS with refractoriness to treatment with nocturnal ventilatory support and emphasize the importance of endoscopic visualization of the upper airway during sleep in order to clarify the origin of refractoriness and concomitantly orient the treatment.

## Consent

Written informed consent was obtained from the patient for publication of this case report and any accompanying images. A copy of the written consent is available for review by the Editor-in-Chief of this journal.

## Competing interests

The authors declare that they have no competing interests.

## Authors' contributions

CL analyzed and interpreted the patient data regarding OSAS and reviewed the existing literature on this issue. MD also analyzed and interpreted the patient data and was a major contributor in writing the manuscript. AM performed the sleep nasoendoscopy. MG performed the adaptation to noninvasive ventilation. ESC analyzed the polyssonographic data. JW orientated the investigation and therapeutic options and was a major contributor in writing the manuscript. All authors read and approved the final manuscript.
